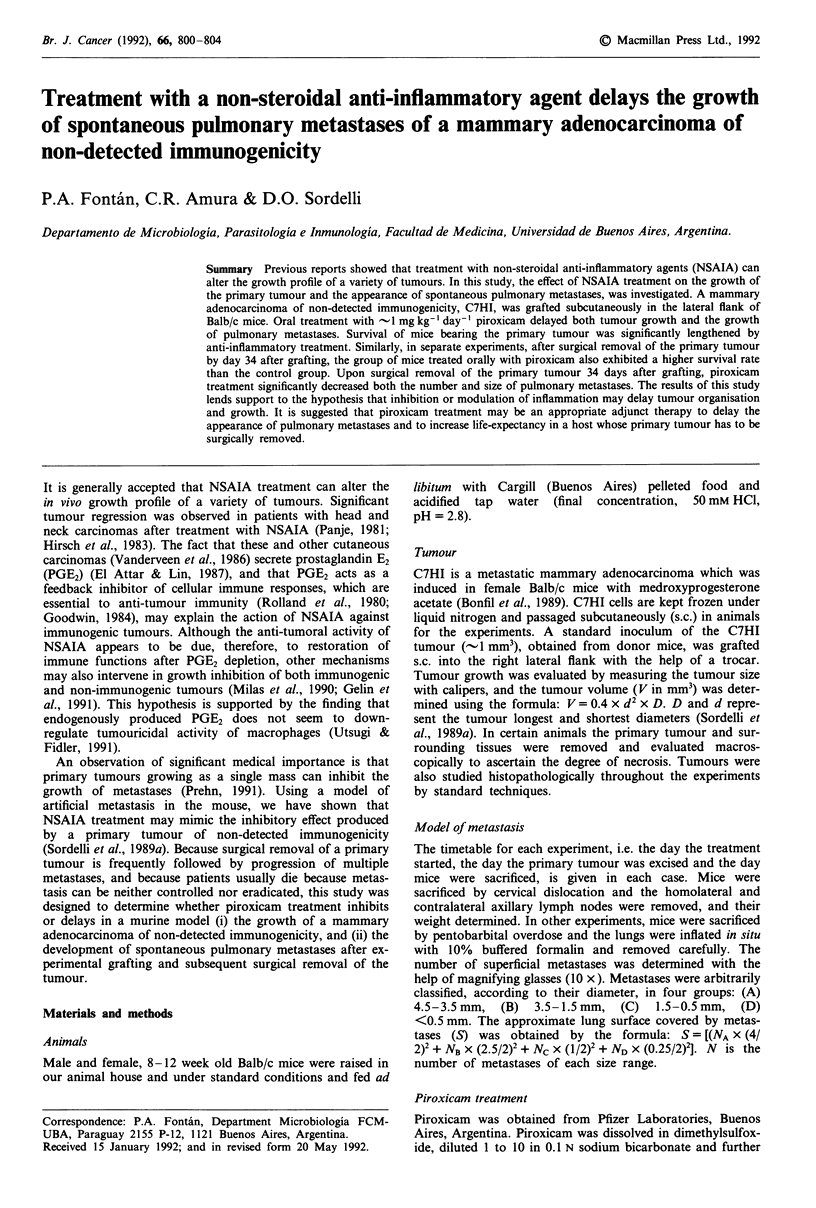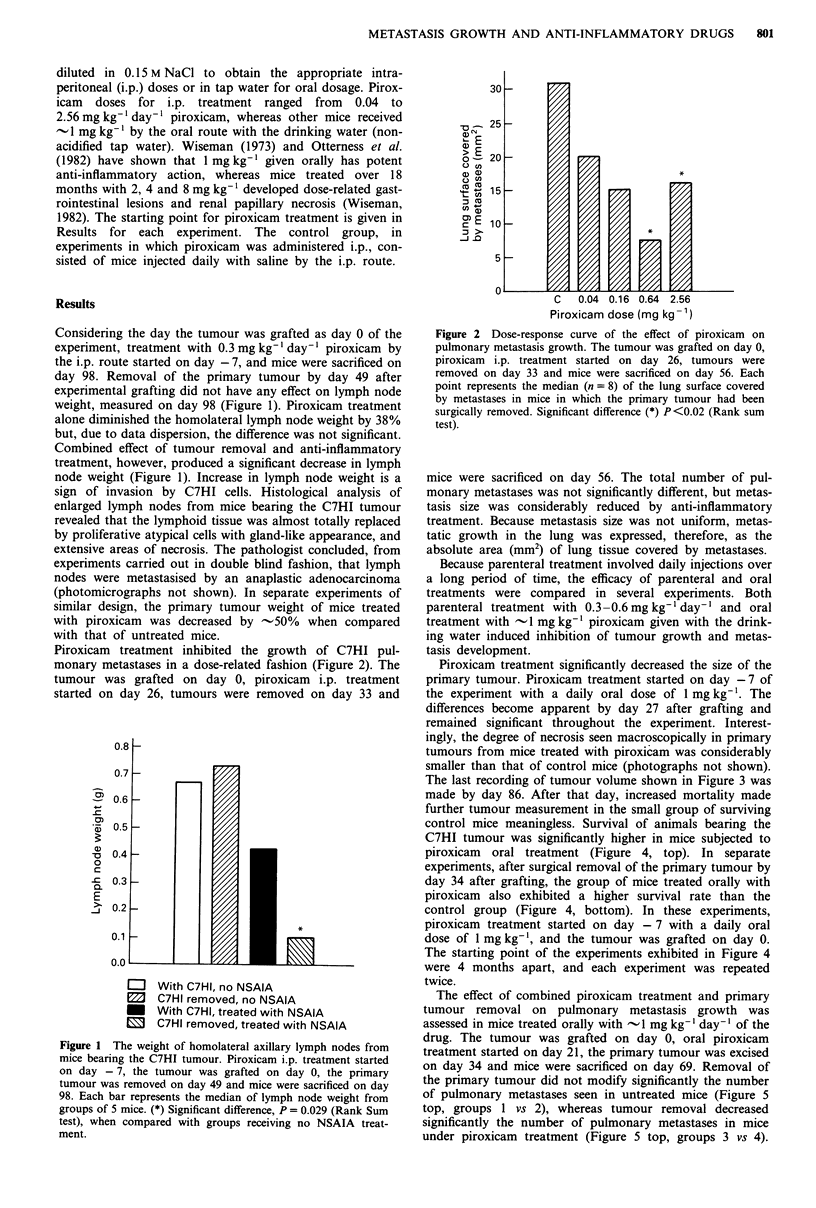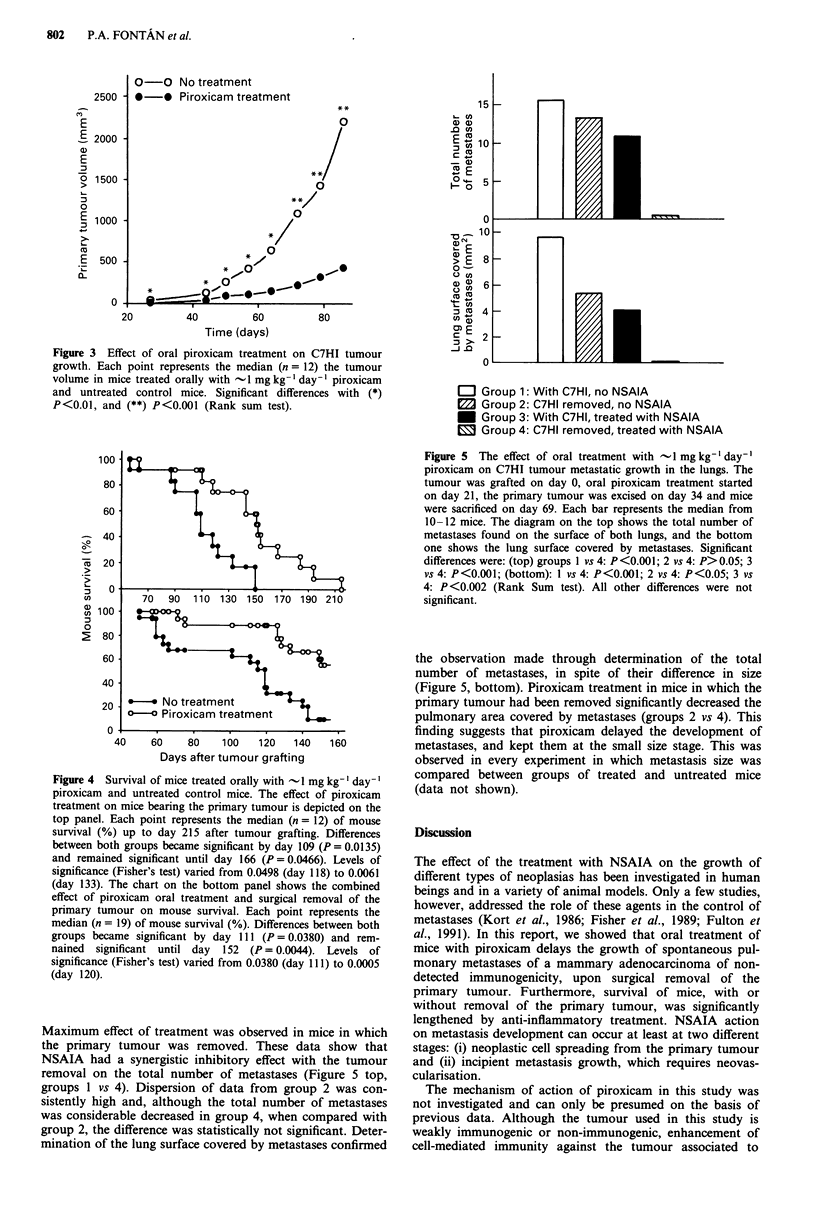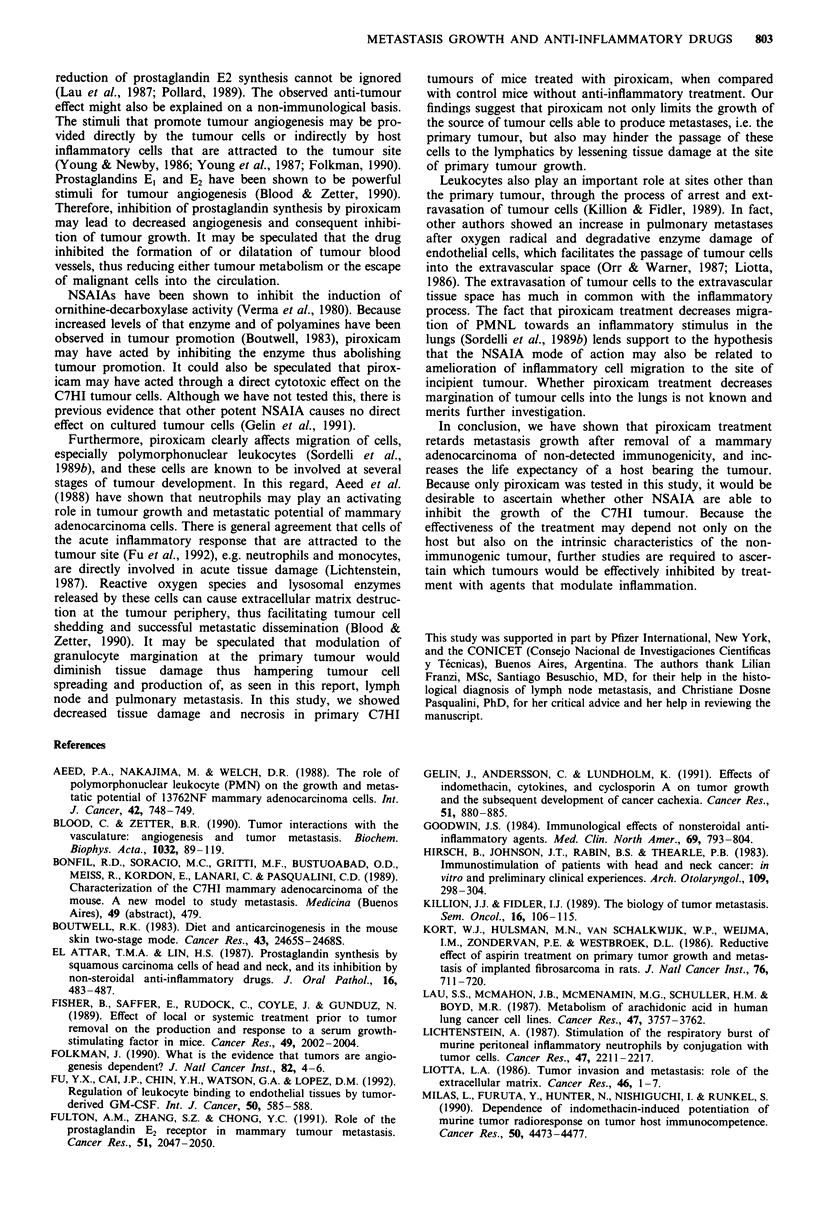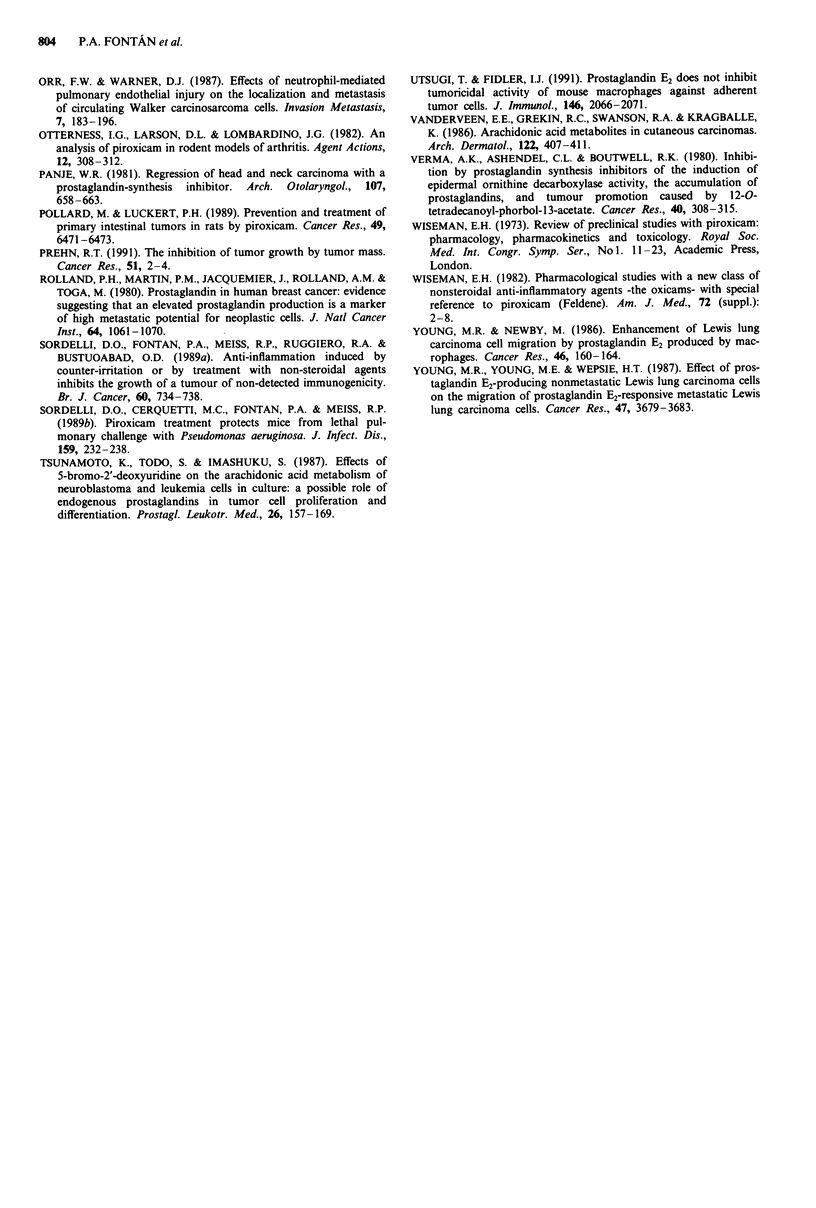# Treatment with a non-steroidal anti-inflammatory agent delays the growth of spontaneous pulmonary metastases of a mammary adenocarcinoma of non-detected immunogenicity.

**DOI:** 10.1038/bjc.1992.363

**Published:** 1992-11

**Authors:** P. A. Fontán, C. R. Amura, D. O. Sordelli

**Affiliations:** Departamento de Microbiología, Parasitología e Inmunología, Facultad de Medicina, Universidad de Buenos Aires, Argentina.

## Abstract

Previous reports showed that treatment with non-steroidal anti-inflammatory agents (NSAIA) can alter the growth profile of a variety of tumours. In this study, the effect of NSAIA treatment on the growth of the primary tumour and the appearance of spontaneous pulmonary metastases, was investigated. A mammary adenocarcinoma of non-detected immunogenicity, C7HI, was grafted subcutaneously in the lateral flank of Balb/c mice. Oral treatment with approximately 1 mg kg-1 day-1 piroxicam delayed both tumour growth and the growth of pulmonary metastases. Survival of mice bearing the primary tumour was significantly lengthened by anti-inflammatory treatment. Similarly, in separate experiments, after surgical removal of the primary tumour by day 34 after grafting, the group of mice treated orally with piroxicam also exhibited a higher survival rate than the control group. Upon surgical removal of the primary tumour 34 days after grafting, piroxicam treatment significantly decreased both the number and size of pulmonary metastases. The results of this study lends support to the hypothesis that inhibition or modulation of inflammation may delay tumour organisation and growth. It is suggested that piroxicam treatment may be an appropriate adjunct therapy to delay the appearance of pulmonary metastases and to increase life-expectancy in a host whose primary tumour has to be surgically removed.


Br. J. Cancer (1992), 66, 800–804 © Macmillan Press Ltd., 1992

### Treatment with a non-steroidal anti-inflammatory agent delays the growth of spontaneous pulmonary metastases of a mammary adenocarcinoma of non-detected immunogenicity

P.A. Fontán, C.R. Amura & D.O. Sordelli

*Departamento de Microbiología, Parasitología e Inmunología, Facultad de Medicina, Universidad de Buenos Aires, Argentina.*

**Summary** Previous reports showed that treatment with non-steroidal anti-inflammatory agents (NSAIA) can alter the growth profile of a variety of tumours. In this study, the effect of NSAIA treatment on the growth of the primary tumour and the appearance of spontaneous pulmonary metastases, was investigated. A mammary adenocarcinoma of non-detected immunogenicity, C7HI, was grafted subcutaneously in the lateral flank of Balb/c mice. Oral treatment with $\sim 1\ mg\ kg^{-1}\ day^{-1}$ piroxicam delayed both tumour growth and the growth of pulmonary metastases. Survival of mice bearing the primary tumour was significantly lengthened by anti-inflammatory treatment. Similarly, in separate experiments, after surgical removal of the primary tumour by day 34 after grafting, the group of mice treated orally with piroxicam also exhibited a higher survival rate than the control group. Upon surgical removal of the primary tumour 34 days after grafting, piroxicam treatment significantly decreased both the number and size of pulmonary metastases. The results of this study lends support to the hypothesis that inhibition or modulation of inflammation may delay tumour organisation and growth. It is suggested that piroxicam treatment may be an appropriate adjunct therapy to delay the appearance of pulmonary metastases and to increase life-expectancy in a host whose primary tumour has to be surgically removed.

It is generally accepted that NSAIA treatment can alter the *in vivo* growth profile of a variety of tumours. Significant tumour regression was observed in patients with head and neck carcinomas after treatment with NSAIA (Panje, 1981; Hirsch *et al.*, 1983). The fact that these and other cutaneous carcinomas (Vanderveen *et al.*, 1986) secrete prostaglandin $E_2$ ($PGE_2$) (El Attar & Lin, 1987), and that $PGE_2$ acts as a feedback inhibitor of cellular immune responses, which are essential to anti-tumour immunity (Rolland *et al.*, 1980; Goodwin, 1984), may explain the action of NSAIA against immunogenic tumours. Although the anti-tumoral activity of NSAIA appears to be due, therefore, to restoration of immune functions after $PGE_2$ depletion, other mechanisms may also intervene in growth inhibition of both immunogenic and non-immunogenic tumours (Milas *et al.*, 1990; Gelin *et al.*, 1991). This hypothesis is supported by the finding that endogenously produced $PGE_2$ does not seem to down-regulate tumouricidal activity of macrophages (Utsugi & Fidler, 1991).

An observation of significant medical importance is that primary tumours growing as a single mass can inhibit the growth of metastases (Prehn, 1991). Using a model of artificial metastasis in the mouse, we have shown that NSAIA treatment may mimic the inhibitory effect produced by a primary tumour of non-detected immunogenicity (Sordelli *et al.*, 1989*a*). Because surgical removal of a primary tumour is frequently followed by progression of multiple metastases, and because patients usually die because metastasis can be neither controlled nor eradicated, this study was designed to determine whether piroxicam treatment inhibits or delays in a murine model (i) the growth of a mammary adenocarcinoma of non-detected immunogenicity, and (ii) the development of spontaneous pulmonary metastases after experimental grafting and subsequent surgical removal of the tumour.

### Materials and methods

### *Animals*

Male and female, 8–12 week old Balb/c mice were raised in our animal house and under standard conditions and fed *ad libitum* with Cargill (Buenos Aires) pelleted food and acidified tap water (final concentration, 50 mM HCl, pH = 2.8).

### *Tumour*

C7HI is a metastatic mammary adenocarcinoma which was induced in female Balb/c mice with medroxyprogesterone acetate (Bonfil *et al.*, 1989). C7HI cells are kept frozen under liquid nitrogen and passaged subcutaneously (s.c.) in animals for the experiments. A standard inoculum of the C7HI tumour ($\sim 1\ mm^3$), obtained from donor mice, was grafted s.c. into the right lateral flank with the help of a trocar. Tumour growth was evaluated by measuring the tumour size with calipers, and the tumour volume ($V$ in $mm^3$) was determined using the formula: $V = 0.4 \times d^2 \times D$. $D$ and $d$ represent the tumour longest and shortest diameters (Sordelli *et al.*, 1989*a*). In certain animals the primary tumour and surrounding tissues were removed and evaluated macroscopically to ascertain the degree of necrosis. Tumours were also studied histopathologically throughout the experiments by standard techniques.

### *Model of metastasis*

The timetable for each experiment, i.e. the day the treatment started, the day the primary tumour was excised and the day mice were sacrificed, is given in each case. Mice were sacrificed by cervical dislocation and the homolateral and contralateral axillary lymph nodes were removed, and their weight determined. In other experiments, mice were sacrificed by pentobarbital overdose and the lungs were inflated *in situ* with 10% buffered formalin and removed carefully. The number of superficial metastases was determined with the help of magnifying glasses (10 ×). Metastases were arbitrarily classified, according to their diameter, in four groups: (A) 4.5–3.5 mm, (B) 3.5–1.5 mm, (C) 1.5–0.5 mm, (D) <0.5 mm. The approximate lung surface covered by metastases ($S$) was obtained by the formula: $S = [(N_A \times (4/2)^2 + N_B \times (2.5/2)^2 + N_C \times (1/2)^2 + N_D \times (0.25/2)^2]$. $N$ is the number of metastases of each size range.

### *Piroxicam treatment*

Piroxicam was obtained from Pfizer Laboratories, Buenos Aires, Argentina. Piroxicam was dissolved in dimethylsulfoxide, diluted 1 to 10 in 0.1 N sodium bicarbonate and further

Correspondence: P.A. Fontán, Department Microbiología FCM-UBA, Paraguay 2155 P-12, 1121 Buenos Aires, Argentina.
Received 15 January 1992; and in revised form 20 May 1992.

diluted in 0.15 M NaCl to obtain the appropriate intraperitoneal (i.p.) doses or in tap water for oral dosage. Piroxicam doses for i.p. treatment ranged from 0.04 to 2.56 mg kg$^{-1}$ day$^{-1}$ piroxicam, whereas other mice received ~1 mg kg$^{-1}$ by the oral route with the drinking water (non-acidified tap water). Wiseman (1973) and Otterness *et al.* (1982) have shown that 1 mg kg$^{-1}$ given orally has potent anti-inflammatory action, whereas mice treated over 18 months with 2, 4 and 8 mg kg$^{-1}$ developed dose-related gastrointestinal lesions and renal papillary necrosis (Wiseman, 1982). The starting point for piroxicam treatment is given in Results for each experiment. The control group, in experiments in which piroxicam was administered i.p., consisted of mice injected daily with saline by the i.p. route.

### Results

Considering the day the tumour was grafted as day 0 of the experiment, treatment with 0.3 mg kg$^{-1}$ day$^{-1}$ piroxicam by the i.p. route started on day −7, and mice were sacrificed on day 98. Removal of the primary tumour by day 49 after experimental grafting did not have any effect on lymph node weight, measured on day 98 (Figure 1). Piroxicam treatment alone diminished the homolateral lymph node weight by 38% but, due to data dispersion, the difference was not significant. Combined effect of tumour removal and anti-inflammatory treatment, however, produced a significant decrease in lymph node weight (Figure 1). Increase in lymph node weight is a sign of invasion by C7HI cells. Histological analysis of enlarged lymph nodes from mice bearing the C7HI tumour revealed that the lymphoid tissue was almost totally replaced by proliferative atypical cells with gland-like appearance, and extensive areas of necrosis. The pathologist concluded, from experiments carried out in double blind fashion, that lymph nodes were metastasised by an anaplastic adenocarcinoma (photomicrographs not shown). In separate experiments of similar design, the primary tumour weight of mice treated with piroxicam was decreased by ~50% when compared with that of untreated mice.

Piroxicam treatment inhibited the growth of C7HI pulmonary metastases in a dose-related fashion (Figure 2). The tumour was grafted on day 0, piroxicam i.p. treatment started on day 26, tumours were removed on day 33 and

**Figure 1** The weight of homolateral axillary lymph nodes from mice bearing the C7HI tumour. Piroxicam i.p. treatment started on day −7, the tumour was grafted on day 0, the primary tumour was removed on day 49 and mice were sacrificed on day 98. Each bar represents the median of lymph node weight from groups of 5 mice. (*) Significant difference, $P = 0.029$ (Rank Sum test), when compared with groups receiving no NSAIA treatment.

**Figure 2** Dose-response curve of the effect of piroxicam on pulmonary metastasis growth. The tumour was grafted on day 0, piroxicam i.p. treatment started on day 26, tumours were removed on day 33 and mice were sacrificed on day 56. Each point represents the median ($n = 8$) of the lung surface covered by metastases in mice in which the primary tumour had been surgically removed. Significant difference (*) $P < 0.02$ (Rank sum test).

mice were sacrificed on day 56. The total number of pulmonary metastases was not significantly different, but metastasis size was considerably reduced by anti-inflammatory treatment. Because metastasis size was not uniform, metastatic growth in the lung was expressed, therefore, as the absolute area (mm$^2$) of lung tissue covered by metastases.

Because parenteral treatment involved daily injections over a long period of time, the efficacy of parenteral and oral treatments were compared in several experiments. Both parenteral treatment with 0.3–0.6 mg kg$^{-1}$ day$^{-1}$ and oral treatment with ~1 mg kg$^{-1}$ piroxicam given with the drinking water induced inhibition of tumour growth and metastasis development.

Piroxicam treatment significantly decreased the size of the primary tumour. Piroxicam treatment started on day −7 of the experiment with a daily oral dose of 1 mg kg$^{-1}$. The differences become apparent by day 27 after grafting and remained significant throughout the experiment. Interestingly, the degree of necrosis seen macroscopically in primary tumours from mice treated with piroxicam was considerably smaller than that of control mice (photographs not shown). The last recording of tumour volume shown in Figure 3 was made by day 86. After that day, increased mortality made further tumour measurement in the small group of surviving control mice meaningless. Survival of animals bearing the C7HI tumour was significantly higher in mice subjected to piroxicam oral treatment (Figure 4, top). In separate experiments, after surgical removal of the primary tumour by day 34 after grafting, the group of mice treated orally with piroxicam also exhibited a higher survival rate than the control group (Figure 4, bottom). In these experiments, piroxicam treatment started on day −7 with a daily oral dose of 1 mg kg$^{-1}$, and the tumour was grafted on day 0. The starting point of the experiments exhibited in Figure 4 were 4 months apart, and each experiment was repeated twice.

The effect of combined piroxicam treatment and primary tumour removal on pulmonary metastasis growth was assessed in mice treated orally with ~1 mg kg$^{-1}$ day$^{-1}$ of the drug. The tumour was grafted on day 0, oral piroxicam treatment started on day 21, the primary tumour was excised on day 34 and mice were sacrificed on day 69. Removal of the primary tumour did not modify significantly the number of pulmonary metastases seen in untreated mice (Figure 5 top, groups 1 *vs* 2), whereas tumour removal decreased significantly the number of pulmonary metastases in mice under piroxicam treatment (Figure 5 top, groups 3 *vs* 4).

Figure 3 Effect of oral piroxicam treatment on C7HI tumour growth. Each point represents the median ($n = 12$) the tumour volume in mice treated orally with ~1 mg kg$^{-1}$ day$^{-1}$ piroxicam and untreated control mice. Significant differences with (*) $P<0.01$, and (**) $P<0.001$ (Rank sum test).

Figure 4 Survival of mice treated orally with ~1 mg kg$^{-1}$ day$^{-1}$ piroxicam and untreated control mice. The effect of piroxicam treatment on mice bearing the primary tumour is depicted on the top panel. Each point represents the median ($n = 12$) of mouse survival (%) up to day 215 after tumour grafting. Differences between both groups became significant by day 109 ($P = 0.0135$) and remained significant until day 166 ($P = 0.0466$). Levels of significance (Fisher's test) varied from 0.0498 (day 118) to 0.0061 (day 133). The chart on the bottom panel shows the combined effect of piroxicam oral treatment and surgical removal of the primary tumour on mouse survival. Each point represents the median ($n = 19$) of mouse survival (%). Differences between both groups became significant by day 111 ($P = 0.0380$) and remnained significant until day 152 ($P = 0.0044$). Levels of significance (Fisher's test) varied from 0.0380 (day 111) to 0.0005 (day 120).

Maximum effect of treatment was observed in mice in which the primary tumour was removed. These data show that NSAIA had a synergistic inhibitory effect with the tumour removal on the total number of metastases (Figure 5 top, groups 1 *vs* 4). Dispersion of data from group 2 was consistently high and, although the total number of metastases was considerable decreased in group 4, when compared with group 2, the difference was statistically not significant. Determination of the lung surface covered by metastases confirmed

Figure 5 The effect of oral treatment with ~1 mg kg$^{-1}$ day$^{-1}$ piroxicam on C7HI tumour metastatic growth in the lungs. The tumour was grafted on day 0, oral piroxicam treatment started on day 21, the primary tumour was excised on day 34 and mice were sacrificed on day 69. Each bar represents the median from 10–12 mice. The diagram on the top shows the total number of metastases found on the surface of both lungs, and the bottom one shows the lung surface covered by metastases. Significant differences were: (top) groups 1 *vs* 4: $P<0.001$; 2 *vs* 4: $P>0.05$; 3 *vs* 4: $P<0.001$; (bottom): 1 *vs* 4: $P<0.001$; 2 *vs* 4: $P<0.05$; 3 *vs* 4: $P<0.002$ (Rank Sum test). All other differences were not significant.

the observation made through determination of the total number of metastases, in spite of their difference in size (Figure 5, bottom). Piroxicam treatment in mice in which the primary tumour had been removed significantly decreased the pulmonary area covered by metastases (groups 2 *vs* 4). This finding suggests that piroxicam delayed the development of metastases, and kept them at the small size stage. This was observed in every experiment in which metastasis size was compared between groups of treated and untreated mice (data not shown).

### Discussion

The effect of the treatment with NSAIA on the growth of different types of neoplasias has been investigated in human beings and in a variety of animal models. Only a few studies, however, addressed the role of these agents in the control of metastases (Kort *et al.*, 1986; Fisher *et al.*, 1989; Fulton *et al.*, 1991). In this report, we showed that oral treatment of mice with piroxicam delays the growth of spontaneous pulmonary metastases of a mammary adenocarcinoma of non-detected immunogenicity, upon surgical removal of the primary tumour. Furthermore, survival of mice, with or without removal of the primary tumour, was significantly lengthened by anti-inflammatory treatment. NSAIA action on metastasis development can occur at least at two different stages: (i) neoplastic cell spreading from the primary tumour and (ii) incipient metastasis growth, which requires neovascularisation.

The mechanism of action of piroxicam in this study was not investigated and can only be presumed on the basis of previous data. Although the tumour used in this study is weakly immunogenic or non-immunogenic, enhancement of cell-mediated immunity against the tumour associated to reduction of prostaglandin E2 synthesis cannot be ignored (Lau *et al.*, 1987; Pollard, 1989). The observed anti-tumour effect might also be explained on a non-immunological basis. The stimuli that promote tumour angiogenesis may be provided directly by the tumour cells or indirectly by host inflammatory cells that are attracted to the tumour site (Young & Newby, 1986; Young *et al.*, 1987; Folkman, 1990). Prostaglandins $E_1$ and $E_2$ have been shown to be powerful stimuli for tumour angiogenesis (Blood & Zetter, 1990). Therefore, inhibition of prostaglandin synthesis by piroxicam may lead to decreased angiogenesis and consequent inhibition of tumour growth. It may be speculated that the drug inhibited the formation of or dilatation of tumour blood vessels, thus reducing either tumour metabolism or the escape of malignant cells into the circulation.

NSAIAs have been shown to inhibit the induction of ornithine-decarboxylase activity (Verma *et al.*, 1980). Because increased levels of that enzyme and of polyamines have been observed in tumour promotion (Boutwell, 1983), piroxicam may have acted by inhibiting the enzyme thus abolishing tumour promotion. It could also be speculated that piroxicam may have acted through a direct cytotoxic effect on the C7HI tumour cells. Although we have not tested this, there is previous evidence that other potent NSAIA causes no direct effect on cultured tumour cells (Gelin *et al.*, 1991).

Furthermore, piroxicam clearly affects migration of cells, especially polymorphonuclear leukocytes (Sordelli *et al.*, 1989*b*), and these cells are known to be involved at several stages of tumour development. In this regard, Aeed *et al.* (1988) have shown that neutrophils may play an activating role in tumour growth and metastatic potential of mammary adenocarcinoma cells. There is general agreement that cells of the acute inflammatory response that are attracted to the tumour site (Fu *et al.*, 1992), e.g. neutrophils and monocytes, are directly involved in acute tissue damage (Lichtenstein, 1987). Reactive oxygen species and lysosomal enzymes released by these cells can cause extracellular matrix destruction at the tumour periphery, thus facilitating tumour cell shedding and successful metastatic dissemination (Blood & Zetter, 1990). It may be speculated that modulation of granulocyte margination at the primary tumour would diminish tissue damage thus hampering tumour cell spreading and production of, as seen in this report, lymph node and pulmonary metastasis. In this study, we showed decreased tissue damage and necrosis in primary C7HI tumours of mice treated with piroxicam, when compared with control mice without anti-inflammatory treatment. Our findings suggest that piroxicam not only limits the growth of the source of tumour cells able to produce metastases, i.e. the primary tumour, but also may hinder the passage of these cells to the lymphatics by lessening tissue damage at the site of primary tumour growth.

Leukocytes also play an important role at sites other than the primary tumour, through the process of arrest and extravasation of tumour cells (Killion & Fidler, 1989). In fact, other authors showed an increase in pulmonary metastases after oxygen radical and degradative enzyme damage of endothelial cells, which facilitates the passage of tumour cells into the extravascular space (Orr & Warner, 1987; Liotta, 1986). The extravasation of tumour cells to the extravascular tissue space has much in common with the inflammatory process. The fact that piroxicam treatment decreases migration of PMNL towards an inflammatory stimulus in the lungs (Sordelli *et al.*, 1989*b*) lends support to the hypothesis that the NSAIA mode of action may also be related to amelioration of inflammatory cell migration to the site of incipient tumour. Whether piroxicam treatment decreases margination of tumour cells into the lungs is not known and merits further investigation.

In conclusion, we have shown that piroxicam treatment retards metastasis growth after removal of a mammary adenocarcinoma of non-detected immunogenicity, and increases the life expectancy of a host bearing the tumour. Because only piroxicam was tested in this study, it would be desirable to ascertain whether other NSAIA are able to inhibit the growth of the C7HI tumour. Because the effectiveness of the treatment may depend not only on the host but also on the intrinsic characteristics of the non-immunogenic tumour, further studies are required to ascertain which tumours would be effectively inhibited by treatment with agents that modulate inflammation.

This study was supported in part by Pfizer International, New York, and the CONICET (Consejo Nacional de Investigaciones Científicas y Técnicas), Buenos Aires, Argentina. The authors thank Lilian Franzi, MSc, Santiago Besuschio, MD, for their help in the histological diagnosis of lymph node metastasis, and Christiane Dosne Pasqualini, PhD, for her critical advice and her help in reviewing the manuscript.

### References

AEED, P.A., NAKAJIMA, M. & WELCH, D.R. (1988). The role of polymorphonuclear leukocyte (PMN) on the growth and metastatic potential of 13762NF mammary adenocarcinoma cells. *Int. J. Cancer*, **42**, 748–749.

BLOOD, C. & ZETTER, B.R. (1990). Tumor interactions with the vasculature: angiogenesis and tumor metastasis. *Biochem. Biophys. Acta.*, **1032**, 89–119.

BONFIL, R.D., SORACIO, M.C., GRITTI, M.F., BUSTUOABAD, O.D., MEISS, R., KORDON, E., LANARI, C. & PASQUALINI, C.D. (1989). Characterization of the C7HI mammary adenocarcinoma of the mouse. A new model to study metastasis. *Medicina* (Buenos Aires), **49** (abstract), 479.

BOUTWELL, R.K. (1983). Diet and anticarcinogenesis in the mouse skin two-stage mode. *Cancer Res.*, **43**, 2465S–2468S.

EL ATTAR, T.M.A. & LIN, H.S. (1987). Prostaglandin synthesis by squamous carcinoma cells of head and neck, and its inhibition by non-steroidal anti-inflammatory drugs. *J. Oral Pathol.*, **16**, 483–487.

FISHER, B., SAFFER, E., RUDOCK, C., COYLE, J. & GUNDUZ, N. (1989). Effect of local or systemic treatment prior to tumor removal on the production and response to a serum growth-stimulating factor in mice. *Cancer Res.*, **49**, 2002–2004.

FOLKMAN, J. (1990). What is the evidence that tumors are angiogenesis dependent? *J. Natl Cancer Inst.*, **82**, 4–6.

FU, Y.X., CAI, J.P., CHIN, Y.H., WATSON, G.A. & LOPEZ, D.M. (1992). Regulation of leukocyte binding to endothelial tissues by tumor-derived GM-CSF. *Int. J. Cancer*, **50**, 585–588.

FULTON, A.M., ZHANG, S.Z. & CHONG, Y.C. (1991). Role of the prostaglandin $E_2$ receptor in mammary tumour metastasis. *Cancer Res.*, **51**, 2047–2050.

GELIN, J., ANDERSSON, C. & LUNDHOLM, K. (1991). Effects of indomethacin, cytokines, and cyclosporin A on tumor growth and the subsequent development of cancer cachexia. *Cancer Res.*, **51**, 880–885.

GOODWIN, J.S. (1984). Immunological effects of nonsteroidal anti-inflammatory agents. *Med. Clin. North Amer.*, **69**, 793–804.

HIRSCH, B., JOHNSON, J.T., RABIN, B.S. & THEARLE, P.B. (1983). Immunostimulation of patients with head and neck cancer: *in vitro* and preliminary clinical experiences. *Arch. Otolaryngol.*, **109**, 298–304.

KILLION, J.J. & FIDLER, I.J. (1989). The biology of tumor metastasis. *Sem. Oncol.*, **16**, 106–115.

KORT, W.J., HULSMAN, M.N., VAN SCHALKWIJK, W.P., WEIJMA, I.M., ZONDERVAN, P.E. & WESTBROEK, D.L. (1986). Reductive effect of aspirin treatment on primary tumor growth and metastasis of implanted fibrosarcoma in rats. *J. Natl Cancer Inst.*, **76**, 711–720.

LAU, S.S., MCMAHON, J.B., MCMENAMIN, M.G., SCHULLER, H.M. & BOYD, M.R. (1987). Metabolism of arachidonic acid in human lung cancer cell lines. *Cancer Res.*, **47**, 3757–3762.

LICHTENSTEIN, A. (1987). Stimulation of the respiratory burst of murine peritoneal inflammatory neutrophils by conjugation with tumor cells. *Cancer Res.*, **47**, 2211–2217.

LIOTTA, L.A. (1986). Tumor invasion and metastasis: role of the extracellular matrix. *Cancer Res.*, **46**, 1–7.

MILAS, L., FURUTA, Y., HUNTER, N., NISHIGUCHI, I. & RUNKEL, S. (1990). Dependence of indomethacin-induced potentiation of murine tumor radioresponse on tumor host immunocompetence. *Cancer Res.*, **50**, 4473–4477.

ORR, F.W. & WARNER, D.J. (1987). Effects of neutrophil-mediated pulmonary endothelial injury on the localization and metastasis of circulating Walker carcinosarcoma cells. *Invasion Metastasis*, **7**, 183–196.

OTTERNESS, I.G., LARSON, D.L. & LOMBARDINO, J.G. (1982). An analysis of piroxicam in rodent models of arthritis. *Agent Actions*, **12**, 308–312.

PANJE, W.R. (1981). Regression of head and neck carcinoma with a prostaglandin-synthesis inhibitor. *Arch. Otolaryngol.*, **107**, 658–663.

POLLARD, M. & LUCKERT, P.H. (1989). Prevention and treatment of primary intestinal tumors in rats by piroxicam. *Cancer Res.*, **49**, 6471–6473.

PREHN, R.T. (1991). The inhibition of tumor growth by tumor mass. *Cancer Res.*, **51**, 2–4.

ROLLAND, P.H., MARTIN, P.M., JACQUEMIER, J., ROLLAND, A.M. & TOGA, M. (1980). Prostaglandin in human breast cancer: evidence suggesting that an elevated prostaglandin production is a marker of high metastatic potential for neoplastic cells. *J. Natl Cancer Inst.*, **64**, 1061–1070.

SORDELLI, D.O., FONTAN, P.A., MEISS, R.P., RUGGIERO, R.A. & BUSTUOABAD, O.D. (1989*a*). Anti-inflammation induced by counter-irritation or by treatment with non-steroidal agents inhibits the growth of a tumour of non-detected immunogenicity. *Br. J. Cancer*, **60**, 734–738.

SORDELLI, D.O., CERQUETTI, M.C., FONTAN, P.A. & MEISS, R.P. (1989*b*). Piroxicam treatment protects mice from lethal pulmonary challenge with *Pseudomonas aeruginosa*. *J. Infect. Dis.*, **159**, 232–238.

TSUNAMOTO, K., TODO, S. & IMASHUKU, S. (1987). Effects of 5-bromo-2′-deoxyuridine on the arachidonic acid metabolism of neuroblastoma and leukemia cells in culture: a possible role of endogenous prostaglandins in tumor cell proliferation and differentiation. *Prostagl. Leukotr. Med.*, **26**, 157–169.

UTSUGI, T. & FIDLER, I.J. (1991). Prostaglandin $E_2$ does not inhibit tumoricidal activity of mouse macrophages against adherent tumor cells. *J. Immunol.*, **146**, 2066–2071.

VANDERVEEN, E.E., GREKIN, R.C., SWANSON, R.A. & KRAGBALLE, K. (1986). Arachidonic acid metabolites in cutaneous carcinomas. *Arch. Dermatol.*, **122**, 407–411.

VERMA, A.K., ASHENDEL, C.L. & BOUTWELL, R.K. (1980). Inhibition by prostaglandin synthesis inhibitors of the induction of epidermal ornithine decarboxylase activity, the accumulation of prostaglandins, and tumour promotion caused by 12-*O*-tetradecanoyl-phorbol-13-acetate. *Cancer Res.*, **40**, 308–315.

WISEMAN, E.H. (1973). Review of preclinical studies with piroxicam: pharmacology, pharmacokinetics and toxicology. *Royal Soc. Med. Int. Congr. Symp. Ser.*, No1. 11–23, Academic Press, London.

WISEMAN, E.H. (1982). Pharmacological studies with a new class of nonsteroidal anti-inflammatory agents -the oxicams- with special reference to piroxicam (Feldene). *Am. J. Med.*, **72** (suppl.): 2–8.

YOUNG, M.R. & NEWBY, M. (1986). Enhancement of Lewis lung carcinoma cell migration by prostaglandin $E_2$ produced by macrophages. *Cancer Res.*, **46**, 160–164.

YOUNG, M.R., YOUNG, M.E. & WEPSIE, H.T. (1987). Effect of prostaglandin $E_2$-producing nonmetastatic Lewis lung carcinoma cells on the migration of prostaglandin $E_2$-responsive metastatic Lewis lung carcinoma cells. *Cancer Res.*, **47**, 3679–3683.